# Chromosome 1 Open Reading Frame 35 Drives Colorectal Cancer Progression by Enhancing Tumor‐Intrinsic Proliferation and CD8^+^ T Cell Suppression

**DOI:** 10.1002/mco2.70707

**Published:** 2026-03-28

**Authors:** Shaosen Zhang, Changjiang Yang, Xunye Xu, Lan Lan, Ziyi He, Jiaoting Chen, Caihong Wang

**Affiliations:** ^1^ Department of Etiology and Carcinogenesis National Cancer Center/National Clinical Research Center/Cancer Hospital Chinese Academy of Medical Sciences (CAMS) and Peking Union Medical College (PUMC) Beijing China; ^2^ Key Laboratory of Cancer Genomic Biology Chinese Academy of Medical Sciences and Peking Union Medical College Beijing China; ^3^ Department of Gastroenterological Surgery Peking University People's Hospital Beijing China; ^4^ Beijing Key Laboratory of Precision Diagnosis Treatment and Translational Research for Colorectal Cancer Peking University People's Hospital Beijing China

**Keywords:** CD8^+^ T cells, chromosome 1 open reading frame 35, colorectal cancer, pyrroline‐5‐carboxylate reductase 2, tumor progression

## Abstract

The functional significance of Chromosome 1 open reading frame 35 (C1orf35) in colorectal cancer (CRC) remains poorly characterized. This study investigates its oncogenic role and underlying mechanisms. We report that C1orf35 is frequently upregulated in CRC clinical specimens, and its elevated expression correlates strongly with advanced tumor stage and serves as an independent prognostic indicator for reduced overall survival. Functional assays, including experiments in patient‐derived organoids, demonstrate that C1orf35 is essential for driving tumor cell proliferation, migration, and expansion. Mechanistically, we identify C1orf35 as an upstream activator of the transcription factor c‐Myc. This activation triggers the transcriptional upregulation of the metabolic enzyme pyrroline‐5‐carboxylate reductase 2 (PYCR2), a key node in proline biosynthesis that facilitates tumor growth. Furthermore, we uncover a distinct, non‐cell‐autonomous function of C1orf35 in shaping the tumor immune microenvironment. Through c‐Myc, C1orf35 impairs the cytotoxic function of tumor‐infiltrating CD8^+^ T cells. This inverse spatial relationship between C1orf35 expression and CD8^+^ T‐cell infiltration is validated by multiplex immunohistochemistry in human CRC tissues. Thus, our work defines C1orf35 as a dual‐function oncoprotein that promotes CRC progression by coordinately enhancing tumor‐intrinsic growth via the c‐Myc/PYCR2 axis and fostering an immune‐suppressive niche. These findings nominate C1orf35 as a promising multi‐faceted therapeutic target and prognostic biomarker in CRC.

## Graphical abstract

This study identifies C1orf35 as a key driver of colorectal cancer progression through a dual mechanism. It promotes tumor‐intrinsic cell proliferation and migration via the c‐Myc/PYCR2 axis, and suppresses anti‐tumor immunity by impairing CD8^+^ T‐cell immune surveillance, nominating it as a novel therapeutic target in colorectal cancer.

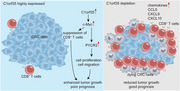

## 1 Introduction

Colorectal cancer (CRC) poses a significant global health challenge as the third most frequent cancer and the second leading cause of cancer death. Annual estimates indicate 1.93 million new cases (about 10% of total cancer diagnoses) and approximately 100,000 fatalities [1, 2]. Despite improvements in traditional treatments such as surgery, radiotherapy, and systemic chemotherapy, the 5‐year survival rate for patients with advanced colorectal cancer remains below 15%. Recently, precision medicine, particularly targeted therapy and immunotherapy, has offered renewed hope for patients with CRC. Nevertheless, their clinical efficacy hinges on a deeper, mechanistic understanding of tumor's molecular mechanisms [3, 4].

Chromosome 1 open reading frame 35 (C1orf35), also known as hMMTAG2, is a gene isolated from the human multiple myeloma (MM) cell line ARH‐77. Its full‐length cDNA spans 1300 bp, is located on chromosome 1q42.13, and contains eight exons, encoding a 263‐amino acid nuclear protein (GenBank: AY137773) [5]. Bioinformatics analysis suggests that the protein is rich in protein kinase phosphorylation and N‐myristoylation sites, and contains nuclear localization signals, implying its potential function as a nuclear signaling molecule involved in transcription or signal transduction. Myelocytomatosis viral oncogene homolog (c‐MYC) has been confirmed to play a key role in various tumors, including CRC, by regulating the cell cycle, metabolic reprogramming, and immune microenvironment remodeling. It not only enhances aerobic glycolysis, but also inhibits p53 activity, thereby promoting chemotherapy resistance and tumor growth [6, 7]. C1orf35 has been identified as a candidate oncogene, binding specifically to the i‐motif in the c‐MYC promoter's NHE III1 region to mediate its regulatory effects, thereby activating c‐MYC transcription [8]. This activation promotes cell‐cycle progression from G1 into S phase and enhances cellular proliferation [9]. C1orf35 is overexpressed in multiple myeloma, gastric cancer, hepatocellular carcinoma, and other malignancies, yet its role in CRC has not been experimentally validated [10]. Thus, the expression pattern, clinical relevance, and underlying molecular mechanisms of C1orf35 in CRC remain incompletely defined. Although immunotherapy has advanced rapidly for CRC treatment [11, 12], the functional impact of C1orf35 on the tumor immune microenvironment (TIME) is still poorly characterized. Given its putative role in remodeling the TIME, C1orf35 therefore represents a candidate target for immunotherapeutic strategies.

Metabolic reprogramming is a well‐established cancer hallmark that supports rapid proliferation, survival, and adaptation to stress. In this context, PYCR2 (pyrroline‐5‐carboxylate reductase 2) serves as the terminal, rate‐limiting enzyme that catalyzes the final step of proline synthesis and has garnered attention for its role in promoting tumor growth. PYCR2 facilitates cellular proliferation, maintains redox homeostasis, and supports mitochondrial function, contributing to metabolic adaptation and survival advantages in various cancers [10, 11]. Notably, our preliminary bioinformatic analyses of CRC datasets revealed a significant positive correlation between the mRNA expression levels of C1orf35 and PYCR2. This compelling correlation suggests a potential functional liaison, prompting the hypothesis that C1orf35 may exert part of its oncogenic effects by modulating PYCR2‐mediated metabolic pathways. Elucidating this putative C1orf35‐PYCR2 axis could bridge a novel, under‐characterized oncogene with an established effector of cancer metabolism, providing a deeper mechanistic understanding of CRC progression.

Given the limited understanding of C1orf35's function in CRC, this study aims to address this gap. First, we integrated transcriptomic data from the Gene Expression Omnibus (GEO) and the Cancer Genome Atlas (TCGA) to compare C1orf35 expression in CRC tumors versus matched normal tissues. We then assessed associations between C1orf35 expression and clinicopathological features and examined its relationship with long‐term survival in an independent clinical cohort to determine its prognostic significance. Next, bioinformatics approaches were employed to explore C1orf35‐related signaling pathways and their interactions within the TIME. Mechanistic experiments further demonstrated that C1orf35 enhances proliferation via the c‐MYC/PYCR2 axis. Additionally, in vitro C1orf35 knockdown models were utilized to evaluate its impact on CRC cell malignant phenotypes. Finally, subcutaneous tumor formation in mice and multiplex immunofluorescence staining of human tissues were employed to validate the regulatory role of C1orf35 in tumor progression and anti‐tumor immunity. These findings aim to provide a novel theoretical framework and potential therapeutic targets for the precise diagnosis and treatment of CRC.

## 2 Results

### 2.1 C1orf35 is Highly Expressed in CRC

**FIGURE 1:**
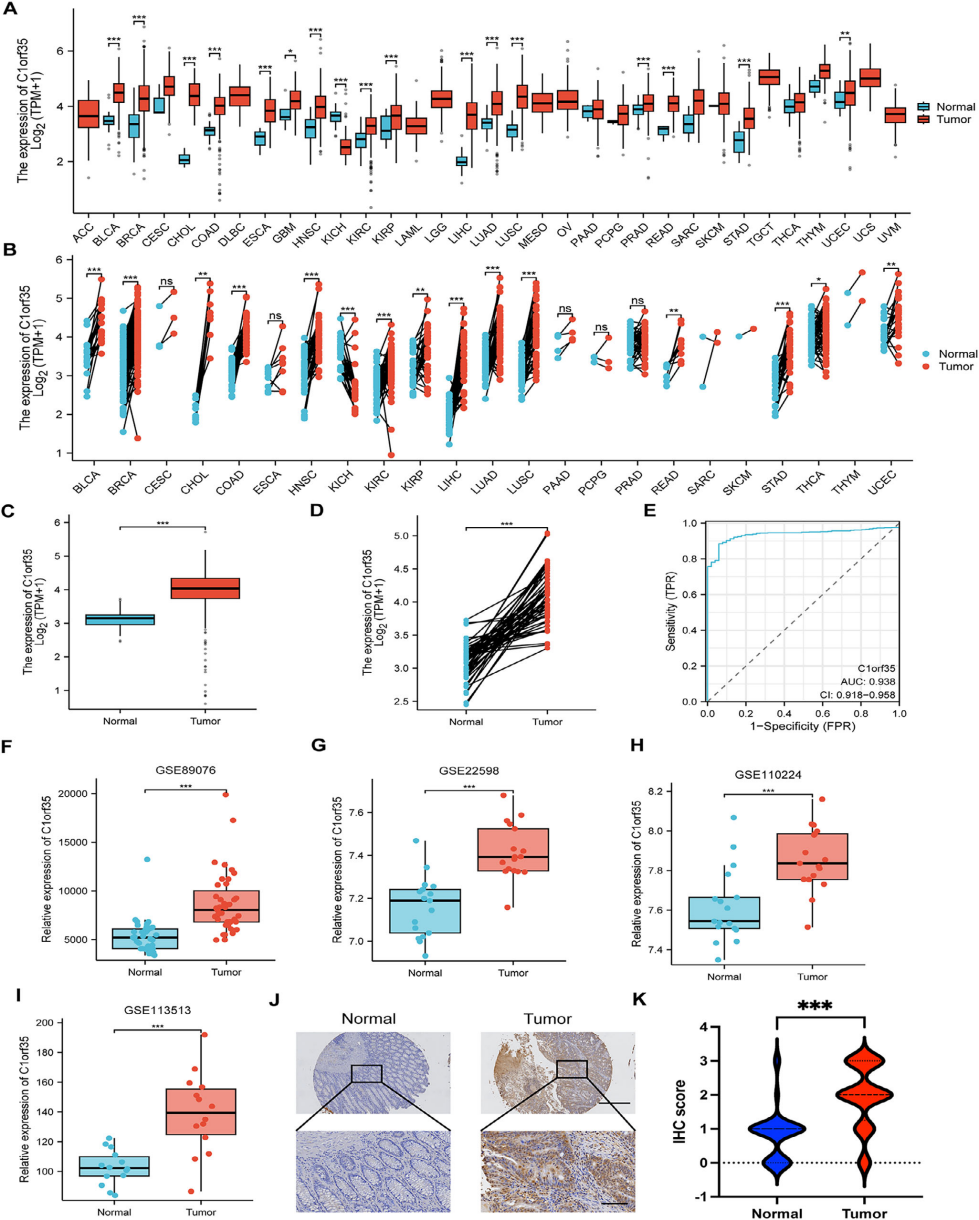
Expression levels of C1orf35 protein and mRNA in pan‐cancer and CRC compared to paired normal samples. (A and B) Pan‐cancer comparison of C1orf35 mRNA levels between tumor and normal tissues using TCGA data. (C) TCGA analysis showing upregulation of C1orf35 mRNA in CRC (*n* = 647) relative to normal mucosa (*n* = 51). (D) TCGA analysis showing upregulation of C1orf35 mRNA in CRC relative to matched normal mucosa (*n* = 50). (E) ROC analysis of C1orf35 in CRC for diagnostic value. (F–I) Validation in GEO cohorts confirming higher C1orf35 mRNA expression in CRC versus matched normal controls in GSE89076 (*n* = 39), GSE22598 (*n* = 17), GSE110224 (*n* = 17), and GSE113513 (*n* = 14). (J and K) IHC staining of C1orf35 in human tumor tissue microarrays (scale bar, 1 mm, 200 µm) (ns indicates no statistical significance, *p < 0.05, **p < 0.01, ***p < 0.001).

To assess the clinical relevance of C1orf35, we examined its expression across tumor and matched normal tissues using TCGA data. C1orf35 mRNA levels differed significantly between malignant and corresponding normal tissues in bladder urothelial carcinoma (BLCA), breast invasive carcinoma (BRCA), cholangiocarcinoma (CHOL), colon adenocarcinoma (COAD), head and neck squamous cell carcinoma (HNSC), chromophobe renal cell carcinoma (KICH), clear cell renal cell carcinoma (KIRC), kidney renal papillary cell carcinoma (KIRP), liver hepatocellular carcinoma (LIHC), lung adenocarcinoma (LUAD), lung squamous cell carcinoma (LUSC), rectum adenocarcinoma (READ), stomach adenocarcinoma (STAD), thyroid carcinoma (THCA) and uterine corpus endometrial carcinoma (UCEC) (Figure 1A,B). The sample sizes for each analysis are provided in the corresponding source data tables (Table S1).

In CRC, TCGA data further confirmed that C1orf35 expression was significantly higher in tumor tissues than in normal tissues (Figure 1C,D). ROC analysis of C1orf35 for distinguishing CRC from normal tissues yielded an AUC of 0.938 (95% CI 0.918–0.958), indicating excellent diagnostic accuracy (Figure 1E). Additional GEO datasets (GSE89076, GSE22598, GSE110224, GSE113513) were analyzed to validate C1orf35 expression, reinforcing these findings (Figure 1F–I). C1orf35 protein expression was further assessed by IHC staining in 92 CRC tumor samples and 45 normal tissues, revealing significantly higher expression levels in tumor tissues compared to normal tissues (Figure 1J,K).

### 2.2 C1orf35 Expression Is Associated With the Pathological Stage of CRC

**FIGURE 2:**
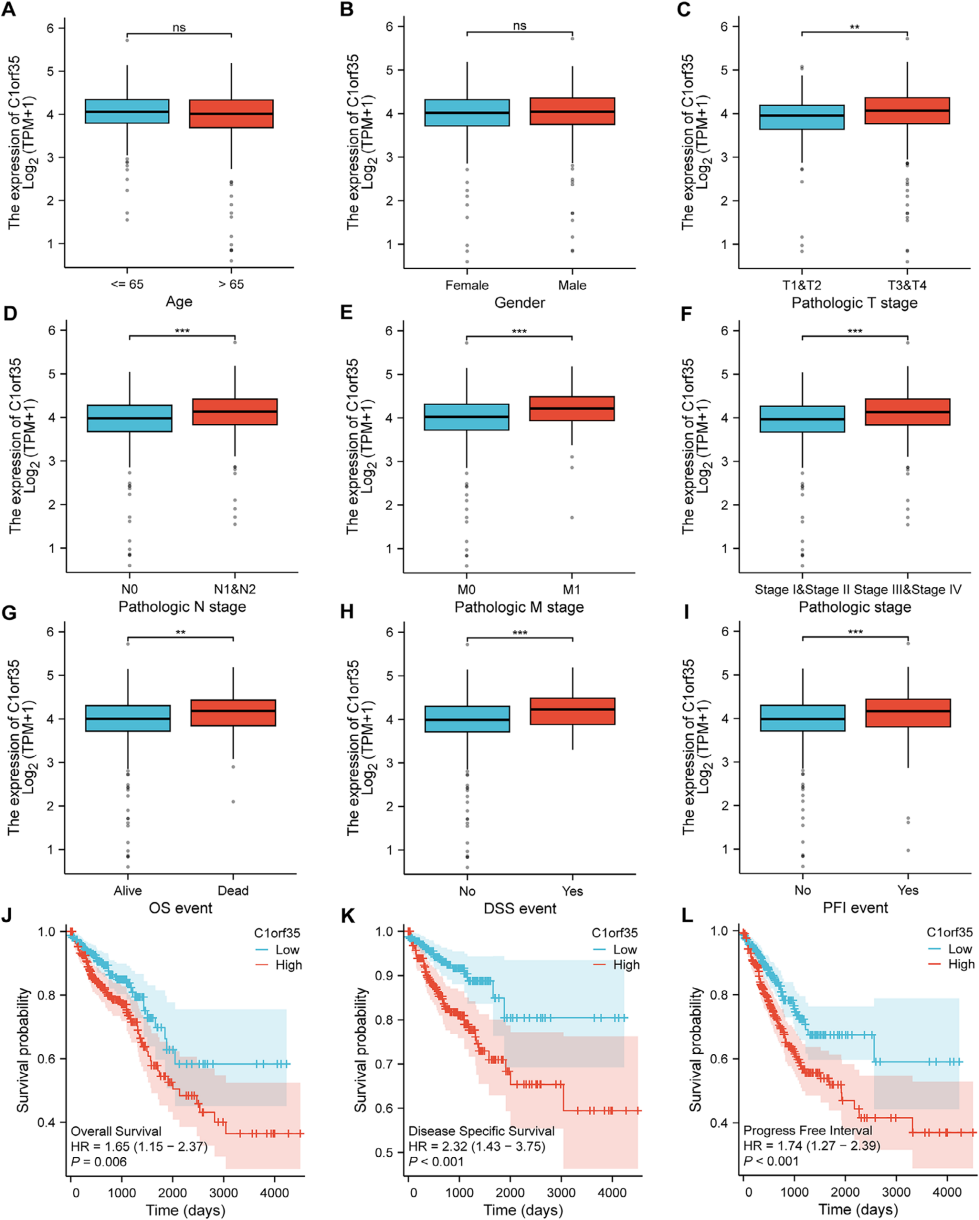
Correlation study of CRC C1orf35 expression with clinical‐pathological parameters and prognosis based on the TCGA database. (A–I) C1orf35 expression was significantly correlated with T stage (C), N stage (D), M stage (E), pathological stage (F), as well as OS (G), DSS (H), and PFI (I). (L) Kaplan–Meier curves showed that upregulated C1orf35 expression was associated with OS, DSS, and PFI (ns indicates no statistical significance, *p < 0.05, **p < 0.01, ***p < 0.001).

The expression characteristics of C1orf35 and its association with clinical‐pathological parameters were systematically analyzed in 644 patients with CRC using the TCGA database. No significant association was observed with patient age or gender (Figure 2A,B). Welch's one‐way ANOVA combined with the Bonferroni post hoc test revealed that C1orf35 expression was significantly positively correlated with tumor T stage, N stage, M stage, and overall pathological stage (*p* < 0.001) (Figure 2C–F). Stratification of patients into high and low C1orf35 expression groups based on the median value indicated that high expression was linked to more advanced T, N, M, and overall TNM stages (Table S2). KM survival curves and subsequent statistical analysis confirmed that elevated C1orf35 expression was associated with significantly shorter overall survival (OS), disease‐specific survival (DSS), and progression‐free interval (PFI) in patients with CRC (Figure 2G–L). These results suggest that C1orf35 could serve as an independent molecular marker reflecting CRC progression and poor prognosis.

### 2.3 C1orf35 Overexpression Is Associated With Poor Prognosis in Patients With CRC

This study systematically evaluated the predictive value of C1orf35 expression for the long‐term prognosis of patients with CRC. KM survival analysis revealed that patients with low C1orf35 expression exhibited significantly better OS, PFI, and DSS compared to those with high expression (Figure 2J–L). Univariate Cox regression analysis identified age > 65 years (*p* < 0.001), T3 and T4 stages (*p* = 0.004), N1 and N2 stages (*p* < 0.001), M1 stage (*p* < 0.001), TNM III/IV stage (*p* < 0.001), and upregulated C1orf35 expression (*p* = 0.006) as significant factors associated with reduced OS. High C1orf35 expression was linked to a 1.65‐fold increase in the risk of death (*p* = 0.006). Multivariate analysis further confirmed that, after adjusting for traditional prognostic factors, C1orf35 remained an independent negative predictor of OS (HR = 1.51, 95% CI 1.01–2.25, *p* = 0.045) (Table S3). Univariate and multivariate Cox regression analyses were performed to evaluate whether C1orf35 expression and clinicopathological variables constitute independent prognostic factors for CRC. In univariate analysis for DSS, T3–T4 stage (*p* = 0.002), N1–N2 stage (*p* < 0.001), M1 stage (*p* < 0.001), overall TNM stage III–IV (*p* < 0.001), and elevated C1orf35 expression (*p* < 0.001) were all significantly associated with poorer DSS.

In the multivariate model, M stage (*p* < 0.001) and TNM III/IV stage (*p* = 0.031) emerged as independent predictors of DSS (Table S4). Regarding PFI, univariate analysis confirmed that T3 and T4 stages (*p* < 0.001), N1 and N2 stages (*p* < 0.001), M1 stage (*p* < 0.001), TNM III/IV stage (*p* < 0.001), and upregulated C1orf35 expression (*p* < 0.001) were significantly associated with a higher risk of recurrence or progression. Multivariate analysis indicated that T3 and T4 stages (*p* < 0.05) and M stage (*p* < 0.001) were independent risk factors for PFI (Table S5). In summary, upregulated C1orf35 expression serves as an independent adverse prognostic marker for OS, DSS, and PFI in patients with CRC, with significant clinical predictive value.

### 2.4 Knockdown of C1orf35 Inhibits CRC Cell Proliferation and Migration

**FIGURE 3:**
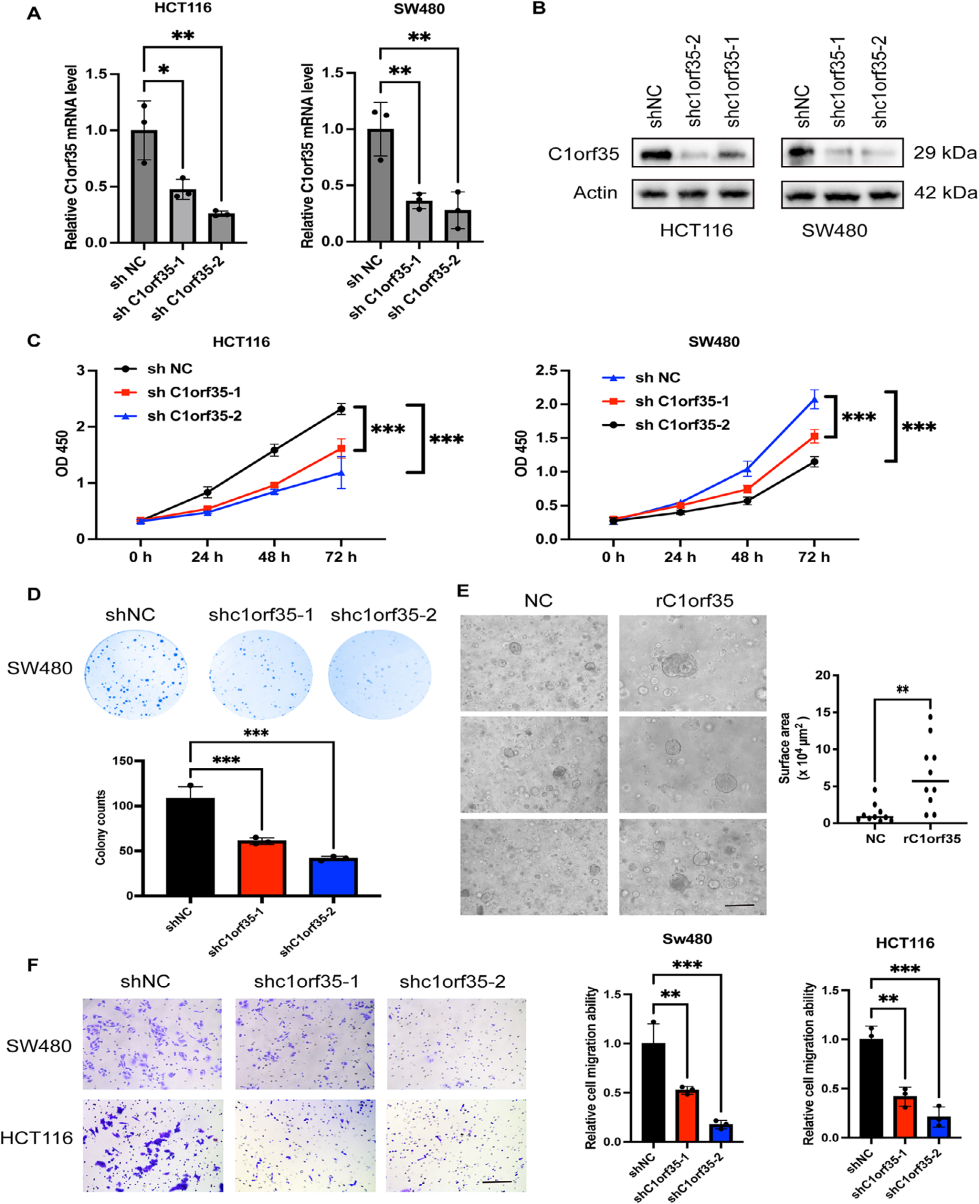
Knockdown of C1orf35 inhibits the proliferation and migration of CRC cells. (A) C1orf35 RNA levels were determined by qRT‐PCR and normalized to actin RNA levels. (B) Western blotting analysis demonstrates decreased C1orf35 levels in C1orf35‐knockdown HCT116 and SW480 cells compared to control cells. (C) Effect of C1orf35 knockdown on cell proliferation in HCT116 and SW480 cells. (D) Colony formation assay in C1orf35‐knockdown SW480 cells. (E) Effect of recombinant C1orf35 protein on CRC organoids (scale bar, 250 µm). (F) Transwell assays in C1orf35‐knockdown SW480 and HCT116 cells (scale bar, 100 µm). Data are presented as mean ± SD; *n* = 3; ns, not significant; *p < 0.05; **p < 0.01; ***p < 0.001; two‐tailed Student's *t*‐tests (E); one‐way ANOVA followed by Dunnett's multiple comparisons (A, C, D, and F).

To investigate the role of C1orf35 in the malignant progression of CRC, two human colon cancer cell lines, HCT116 and SW480, were selected for stable knockdown of C1orf35 expression using lentivirus‐mediated shRNA targeting C1orf35 (shC1orf35). RT‐qPCR and Western blotting confirmed the efficient knockdown of C1orf35 expression (Figure 3A,B). Proliferation assays showed that C1orf35 downregulation significantly inhibited cell proliferation (Figure 3C). Furthermore, colony formation assays demonstrated that C1orf35 knockdown reduced the ability of cells to form colonies, further validating its role in cell proliferation (Figure 3D). Additionally, organoid models derived from patients with CRC were constructed. Exogenous addition of recombinant C1orf35 protein resulted in significantly larger organoids compared to the control group (Figure 3E). C1orf35 knockdown potently inhibited the migratory capacity of colorectal cancer cells, as evidenced by Transwell migration assays (Figure 3F). In conclusion, C1orf35 expression is positively correlated with the proliferative and migratory capabilities of CRC cells.

### 2.5 C1orf35 Promotes Colorectal Cancer Progression Through c‐Myc‐Mediated Upregulation of PYCR2

**FIGURE 4:**
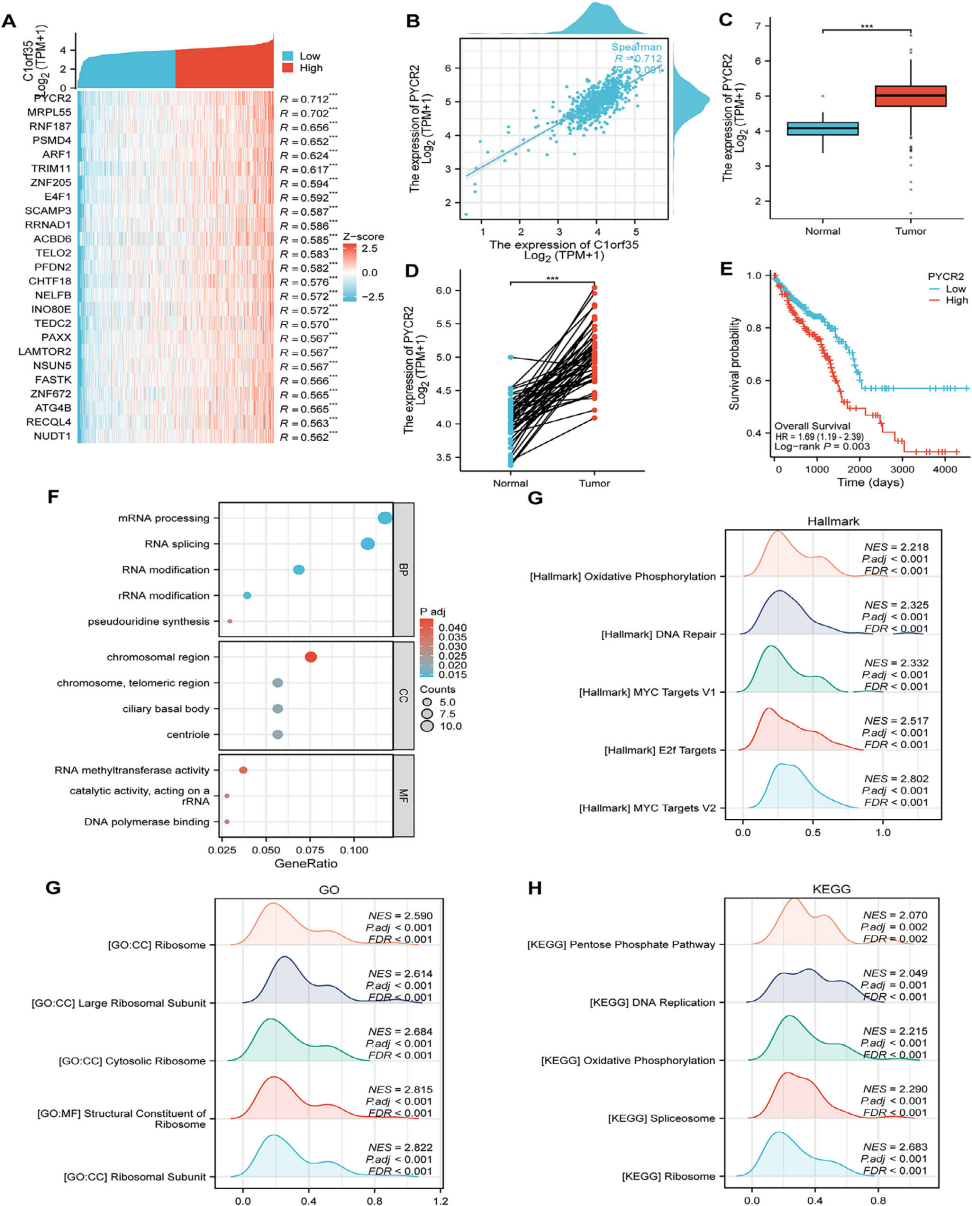
PYCR2 is involved in the oncogenic function of C1orf35 in CRC. (A) Heatmap showing the top 30 genes positively correlated with C1orf35 in CRC. High and low expressions are indicated in red and blue, respectively. (B) Scatter plot of the relationship between C1orf35 and PYCR2 expression levels. (C) TCGA analysis showing upregulation of PYCR2 mRNA in CRC (*n* = 647) relative to normal mucosa (*n* = 51). (D) TCGA analysis showing upregulation of PYCR2 mRNA in CRC relative to matched normal mucosa (*n* = 50). (E) Kaplan–Meier curve showing that upregulation of PYCR2 expression is associated with decreased OS. (F) GO analysis of upregulated and downregulated differentially expressed genes (DEGs). (G–I) Hallmark, GO, and KEGG pathway enrichment analyses of C1orf35‐related genes (ns indicates no significance, *p < 0.05, **p < 0.01, ***p < 0.001).

To systematically investigate the biological function of C1orf35 in CRC, the top 50 genes most strongly correlated with its expression were identified using TCGA data and a heat map was constructed. The results revealed that PYCR2 exhibited the highest correlation coefficient with C1orf35 (Figure 4A). As the terminal and rate‐determining enzyme in proline biosynthesis, PYCR2 catalyzes the NAD(P)H‐dependent conversion of pyrroline‐5‐carboxylate (P5C) to proline. This reaction is fundamental to sustaining cellular homeostasis, influencing redox balance and contributing to oncogenic progression [9]. Previous studies have established that PYCR2 promotes CRC metastasis through multiple signaling pathways and is associated with poor prognosis [13, 14, 15, 16]. These results indicate that PYCR2 may mediate the oncogenic activity of C1orf35.

Further analysis revealed a significant positive correlation between C1orf35 and PYCR2 mRNA levels in CRC samples, and both genes were expressed at significantly higher levels in tumor tissue than in matched normal mucosa (Figure 4B–D). High PYCR2 expression was associated with reduced OS (Figure 4E), corroborating its role as an adverse prognostic marker. We therefore carried out GO, KEGG, and Hallmark enrichment analyses on the gene set correlated with C1orf35 to explore implicated biological processes and pathways. The GO results revealed significant enrichment in “mRNA processing”, “chromosome region”, and “RNA methyltransferase activity”, suggesting a role in promoting DNA replication and cell proliferation through the regulation of mRNA splicing, chromatin remodeling, and telomere homeostasis (Figure 4F). Ribosome‐related pathways were also significantly enriched, further indicating involvement in translational regulation (Figure 4H). Hallmark analysis identified significant activation of oxidative phosphorylation and DNA repair pathways (NES > 2, FDR < 0.001), while KEGG analysis also highlighted the oxidative phosphorylation and DNA replication pathways (Figure 4G,I). These results suggest that C1orf35 may drive the malignant proliferation of CRC by coordinating with PYCR2‐mediated metabolic reprogramming and cell cycle regulation.

**FIGURE 5:**
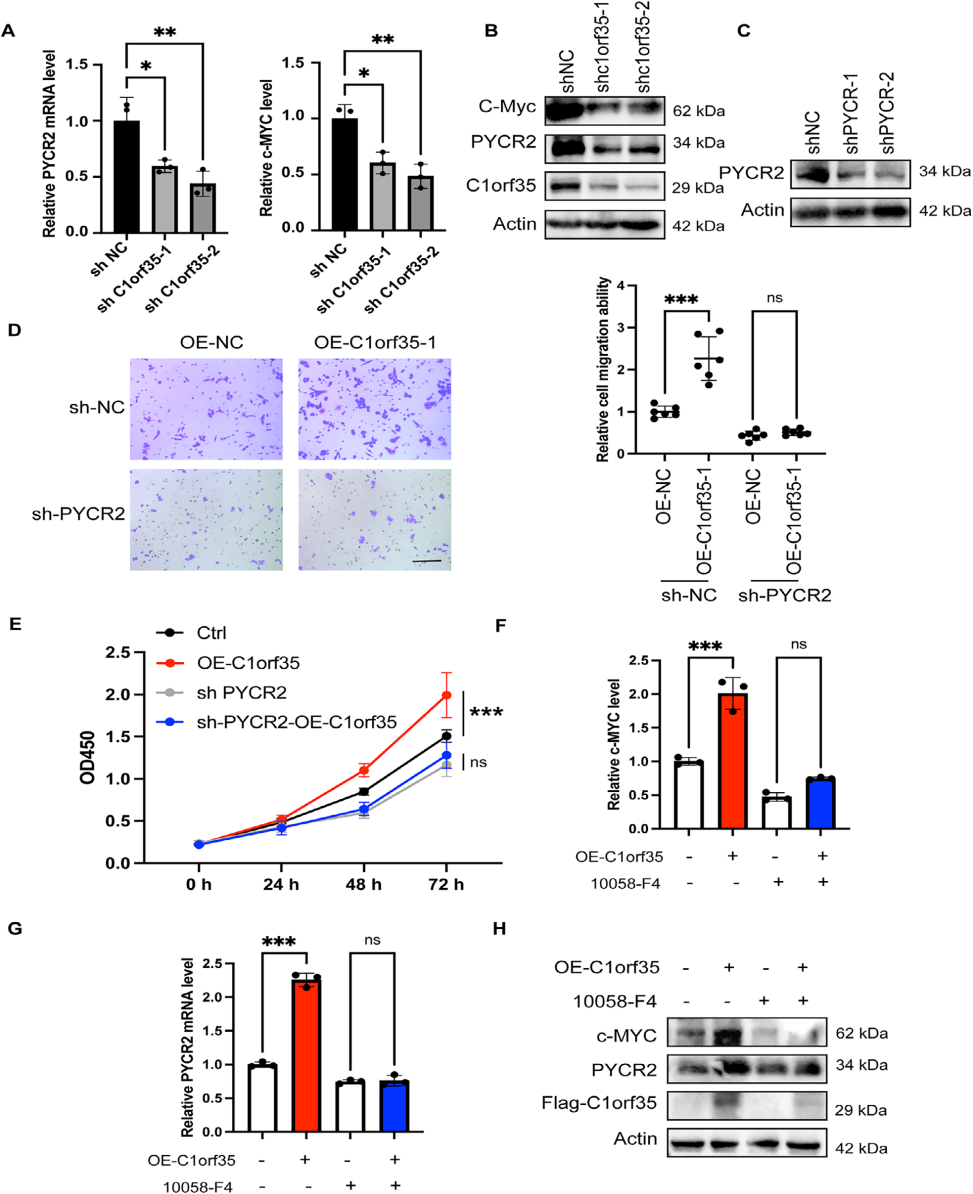
C1orf35 promotes colorectal cancer progression via the c‐Myc/PYCR2 axis. (A) Quantitative RT‐qPCR analysis of c‐Myc and PYCR2 expression in control (shCtrl) and C1orf35‐knockdown (shC1orf35) CRC cells. (B) Western blot analysis of c‐Myc and PYCR2 expression in control (shCtrl) and C1orf35‐knockdown (shC1orf35) CRC cells. (C) Western blotting analysis demonstrates decreased PYCR2 level in PYCR2‐knockdown SW480 cells compared to control cells. (D) PYCR2 is essential for the oncogenic functions of C1orf35. Cell migration was assessed by transwell assay in four groups: control (Ctrl), C1orf35 overexpression (OE), PYCR2 knockdown (shPYCR2), and combined C1orf35 OE + PYCR2 knockdown. Representative images (scale bar, 100 µm) and quantification are shown. (E) Cell proliferation was measured by CCK‐8 assay in the same four groups over 72 h. (F–H) c‐Myc is required for C1orf35‐mediated upregulation of PYCR2. CRC cells were transfected with vector or C1orf35 overexpression plasmid and treated with DMSO or a c‐Myc inhibitor (10058‐F4). Representative RT‐qPCR analysis and western blot of c‐Myc and PYCR2 expression. Data are mean ± SD; *n* = 3; ns, not significant, *p < 0.05, **p < 0.01, ***p < 0.001 (two‐way ANOVA).

To delineate the functional pathway downstream of C1orf35, we first examined its effect on its candidate downstream target PYCR2. Knockdown of C1orf35 in SW480 cells resulted in a concomitant reduction in PYCR2 expression at the mRNA and protein levels (Figure 5A,B). Furthermore, we determined if PYCR2 is the critical effector responsible for the oncogenic phenotypes. In functional rescue experiments, overexpression of C1orf35 significantly enhanced cell migration and proliferation in control cells. However, this pro‐tumorigenic effect was entirely abrogated upon concurrent knockdown of PYCR2 (Figure 5C–E). We next investigated the mechanism by which C1orf35 regulates PYCR2. Previous studies have shown that C1orf35 can directly transcriptionally regulate c‐Myc [8], and c‐Myc itself is a known key transcriptional regulator of PYCR2 [17]. We found knockdown of C1orf35 in SW480 cells resulted in a reduction of both c‐MYC and PYCR2 expression at the mRNA and protein levels (Figure 5A,B). While overexpression of C1orf35 robustly increased PYCR2 expression, pharmacological inhibition of c‐Myc completely abolished this induction (Figure 5F–H). This demonstrates that c‐Myc activity is essential for C1orf35‐mediated upregulation of PYCR2.

Thus, we define a linear oncogenic pathway in CRC: C1orf35 activates the transcription factor c‐Myc, which in turn upregulates PYCR2. The resultant pro‑tumorigenic phenotypes—enhanced proliferation and migration—are strictly dependent on PYCR2, identifying it as the indispensable downstream effector of the C1orf35‑initiated malignant program.

### 2.6 C1orf35 High Expression Is Associated With Low Immune Cell Infiltration in CRC

**FIGURE 6:**
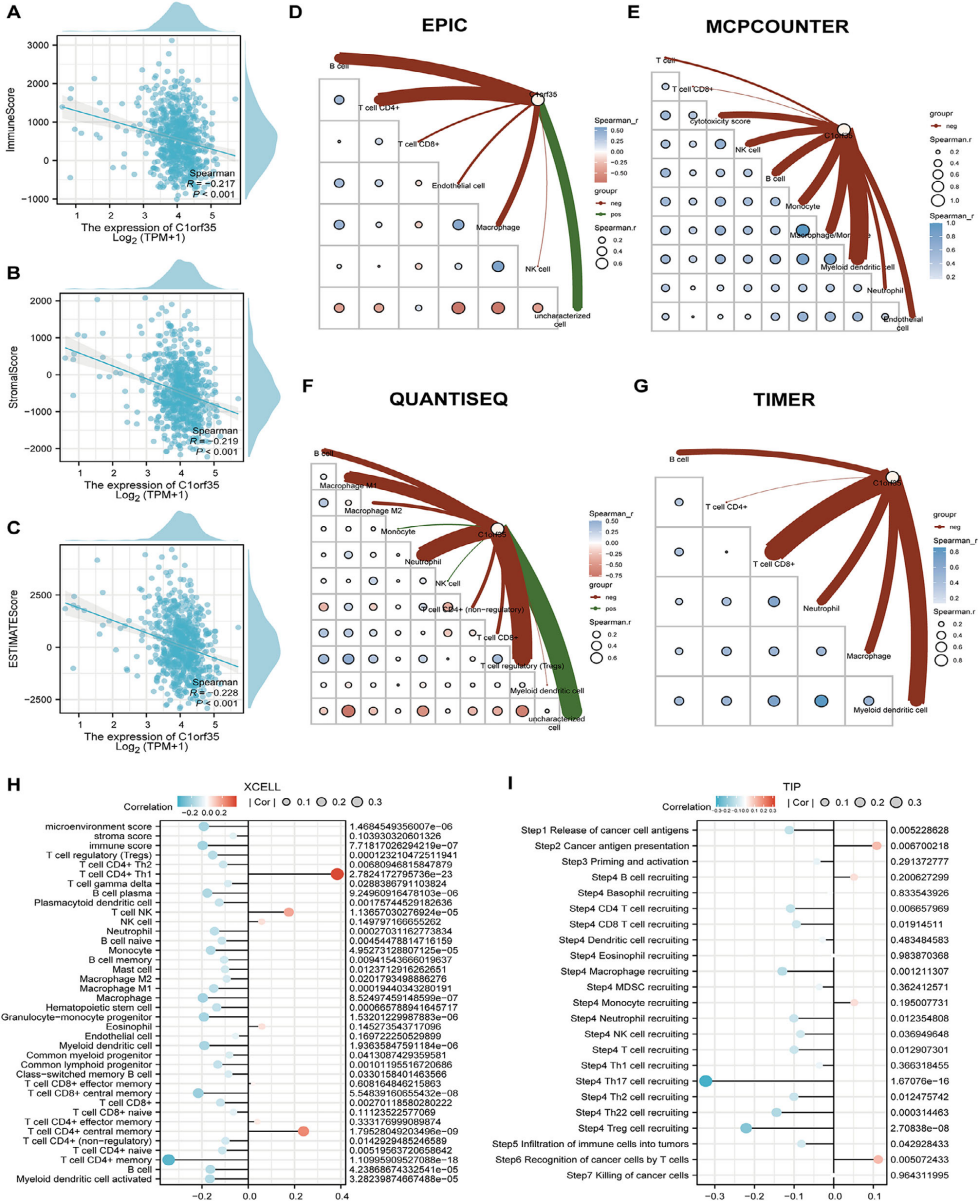
Associations between C1orf35 expression and immune parameters, immune‐cell infiltration, and the tumor microenvironment in colorectal cancer (CRC). (A–C) Correlation of C1orf35 expression with ESTIMATE, stromal, and immune scores. (D) The relationship between C1orf35 expression and immune cell infiltration in CRC based on EPIC analysis. (E) The relationship between C1orf35 expression and immune cell infiltration in CRC based on MCPCOUNTER analysis. (F) The relationship between C1orf35 expression and immune cell infiltration in CRC based on QUANTISEQ analysis. (G) The relationship between C1orf35 expression and immune cell infiltration in CRC based on TIMER analysis. (H) XCELL‐based associations between C1orf35 expression and the estimated abundance of specific infiltrating immune and stromal cell populations. (I) TIP analysis showing the relationship between C1orf35 expression and functional anti‐tumor immune processes and immune‐cell recruitment in CRC (ns indicates no significance, *p < 0.05, **p < 0.01, ***p < 0.001).

Analysis of the tumor microenvironment (TME) in CRC revealed that the high‐C1orf35 expression group exhibited significantly lower immune, stromal, and ESTIMATE scores compared to the low‐expression group, indicating a strong negative correlation between C1orf35 expression and these TME scores (Figure 6A–C). Using multiple immune deconvolution methods (EPIC, MCPCOUNTER, QUANTISEQ, TIMER, XCELL, and TIP), Spearman correlation analysis further demonstrated that elevated C1orf35 expression inversely associated with reduced infiltration of multiple anti‐tumor immune cell populations within the TME (Table S6). Specifically, EPIC analysis indicated that C1orf35 expression in CRC was negatively correlated with endothelial cells (*r* = −0.081, *p* < 0.05) and macrophages (*r* = −0.130, *p* < 0.05) (Figure 6D). MCPCOUNTER revealed significant negative correlations with T cells (*r* = −0.081, *p* < 0.05), cytotoxicity score (*r* = −0.145, *p* < 0.001), neutrophils (*r* = −0.101, *p* < 0.05), and endothelial cells (*r* = −0.135, *p* < 0.001) (Figure 6E). QUANTISEQ analysis further confirmed a negative correlation between C1orf35 expression and various immune cell types, including B cells (*r* = −0.137, *p* < 0.001), M2 macrophages (*r* = −0.104, *p* < 0.05), CD4^+^ T cells (non‐regulatory) (*r* = −0.098, *p* < 0.05), and CD8^+^ T cells (*r* = −0.099, *p* < 0.05) (Figure 6F). TIMER analysis confirmed a negative correlation between C1orf35 expression and the infitration levels of various immune cell types, including B cells, CD8+ T cells, neutrophils, macrophages, and myeloid dendritic cells (Figure 6G). XCELL analysis also showed that C1orf35 expression in CRC was negatively correlated with regulatory T cells (Tregs), CD4^+^ Th2 cells, gamma delta T cells, plasmacytoid dendritic cells, neutrophils, naive and memory B cells, mast cells, M1 and M2 macrophages, hematopoietic stem cells, myeloid and lymphoid progenitors, class‐switched memory B cells, as well as CD8^+^ T cells, CD4^+^ T cells (non‐regulatory), and naive CD4^+^ T cells (all *p* < 0.05, with specific *p*‐values ranging from < 0.05 to < 0.001) (Figure 6H). TIP analysis revealed that higher C1orf35 expression in CRC was associated with attenuation of multiple anti‐tumor immune processes—including cancer‐cell antigen release, antigen presentation, CD4^+^ and CD8^+^ T‐cell activity, macrophage and neutrophil infiltration, NK‐cell activity, T‐cell‐mediated recognition of cancer cells, and recruitment of Th2 and Th22 cells (all *p* < 0.05; Th22 recruitment *p* < 0.001) (Figure 6I).

In summary, high C1orf35 expression in CRC is closely associated with reduced infiltration of nearly all major immune cell subsets in the TME. These results suggest that C1orf35 may facilitate tumor immune escape and malignant progression by reshaping the immune microenvironment, impairing the host's immunosurveillance and hindering the clearance of tumor cells.

### 2.7 C1orf35 Suppresses CD8^+^ T‐Cell Cytotoxicity and Underlies Their Reduced Infiltration in Colorectal Cancer

**FIGURE 7:**
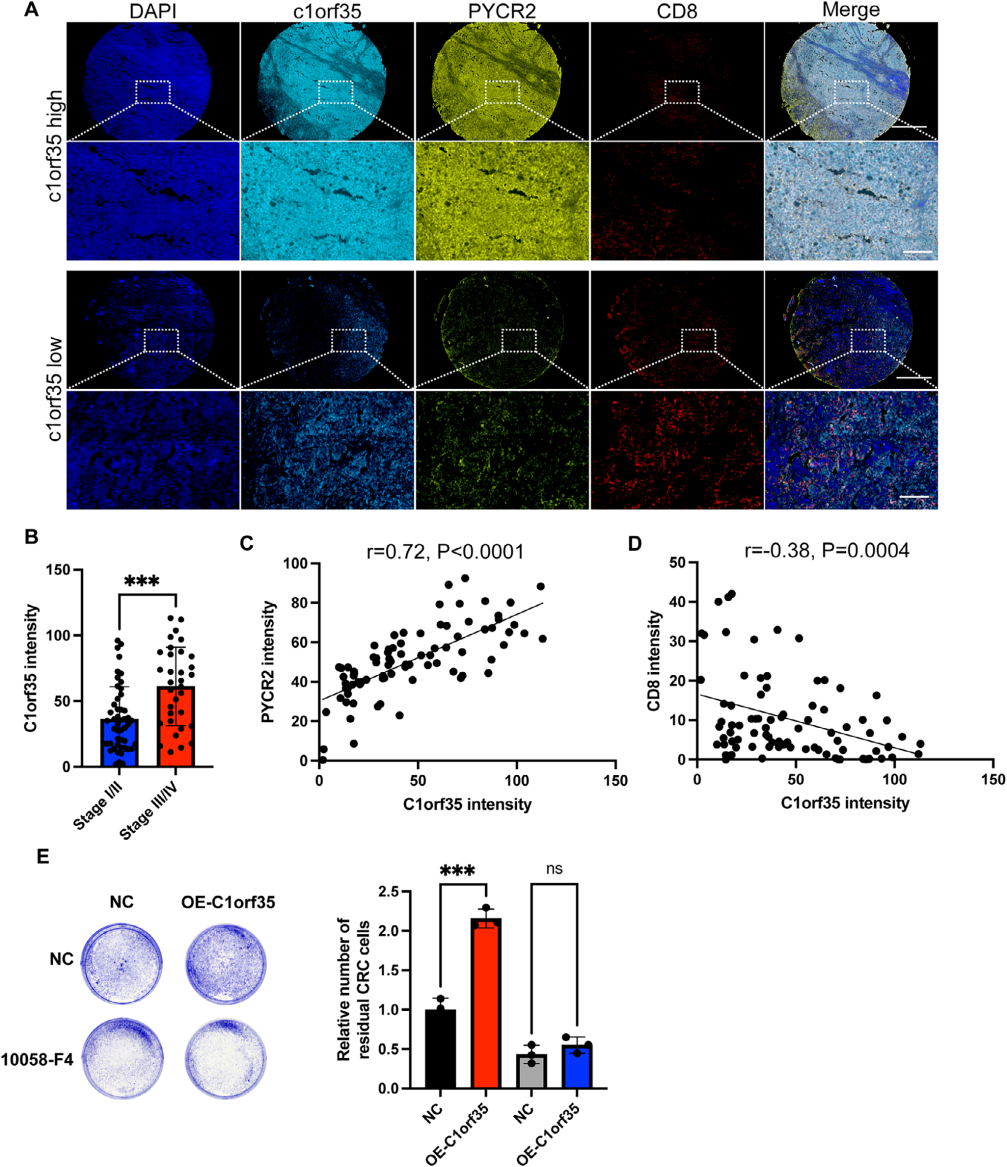
Elevated C1orf35 in human CRC tumor tissues is negatively associated with CD8^+^ T cells. (A) Multiplex immunofluorescence of human tumor tissues (scale bar, 1 mm, 200 µm). (B) Comparison of C1orf35 expression levels in patients with CRC at different stages. (C) Relationship between C1orf35 expression levels and PYCR2 expression levels. (D) Relationship between C1orf35 expression levels and CD8^+^ T‐cell content. (E) C1orf35 impairs CD8^+^ T cell cytotoxicity in a manner dependent on c‐Myc activity. CD8+ T cells were co‐cultured for 24 h with control MC38 cells or MC38 cells overexpressing C1orf35 (OE‐C1orf35) in the presence or absence of the c‐Myc inhibitor 10058‐F4 (10 µM). Tumor cell killing was assessed by crystal violet staining of the remaining adherent MC38 cells. (Left) Representative images. (Right) Quantitative analysis of crystal violet absorbance. Data are mean ± SD (*n* = 3). Statistical significance was determined by two‐way ANOVA (ns, not significant, *p < 0.05, **p < 0.01, ***p < 0.001).

To systematically evaluate the clinical relevance of C1orf35 expression in the malignant progression of CRC, multiplex immunofluorescence staining was performed on 92 CRC tissue samples to analyze C1orf35 and CD8 expression. The results revealed that regions with high C1orf35 expression exhibited significantly enhanced Ki‐67 signals, indicating increased proliferative activity. Concurrently, a higher fluorescence intensity of PYCR2 was observed, confirming the positive correlation between C1orf35 and PYCR2 at the protein level (Figure 7A). In the same field of view, the density of CD8^+^ T cells was significantly reduced, suggesting that high C1orf35 expression may impair local anti‐tumor immunity and be associated with poorer prognosis. When the samples were re‐grouped according to TNM staging, patients with advanced‐stage tumors (Stage III–IV) exhibited significantly elevated C1orf35 expression compared to those with early‐stage disease (Stage I–II) (*p* < 0.001), supporting its involvement in CRC aggressiveness (Figure 7B). Correlation analysis further confirmed that C1orf35 was strongly positively correlated with PYCR2 (*r* = 0.72, *p* < 0.001) and negatively correlated with CD8^+^ T‐cell infiltration (*r* = −0.68, *p* < 0.001) (Figure 7C,D). To functionally assess the impact of C1orf35 on anti‐tumor immunity, we performed a co‐culture assay and found that C1orf35 overexpression in MC38 cells significantly impaired the cytotoxicity of CD8^+^ T cells, an effect that was rescued by treatment with the c‐Myc inhibitor 10058‐F4, demonstrating that C1orf35 suppresses CD8^+^ T‐cell function in a c‐Myc‐dependent manner (Figure 7E). In conclusion, high C1orf35 expression not only reflects increased proliferative capacity and disease progression in CRC but also contributes to an immunosuppressive microenvironment by limiting CD8^+^ T‐cell infiltration. These results suggest that C1orf35 is a promising molecular biomarker indicative of tumor malignancy and immune escape potential in CRC.

### 2.8 Targeting C1orf35 Showed Anti‐tumor Activity in CRC

**FIGURE 8:**
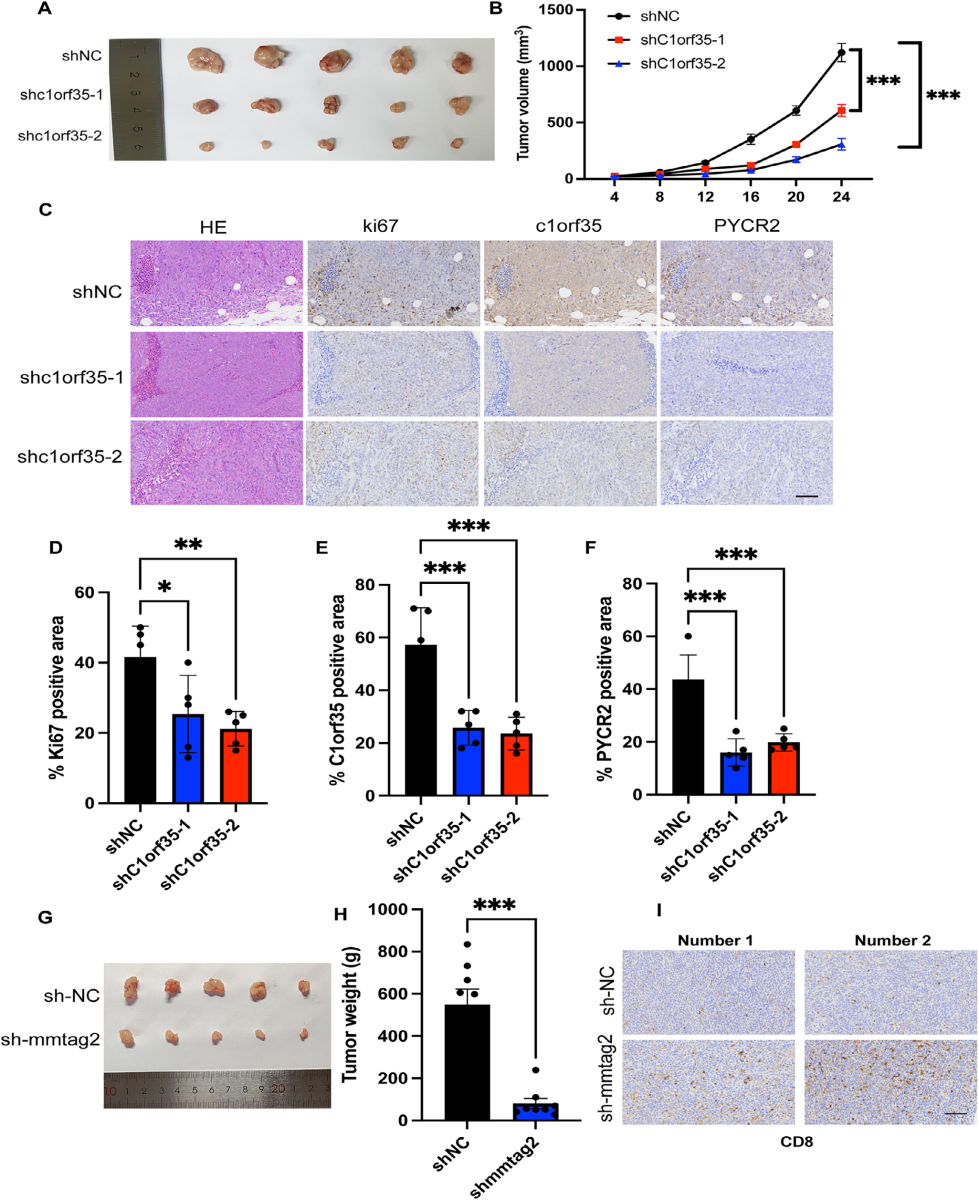
Knockdown of C1orf35 inhibits tumor growth in mice. (A–F) Tumor progression generated by orthotopic injection of HCT116 cells into nude mice (*n* = 7–8/group). (A) Subcutaneous tumor formation experiments with shNC cells, shC1orf35‐1 cells, and shC1orf35‐2 HCT116 cells. (B) Measurement of tumor volume after subcutaneous injection. (C) Immunohistochemical staining results of tumor tissues in the three groups. (D–F) Statistical analysis of immunohistochemical results. Scale bars, 200 µm. (G–I) Tumor progression generated by orthotopic injection of MC38 cells into C57BL/6 mice (n = 7–8/group). (G) Subcutaneous tumor formation experiments with shNC cells, shmmtag2 MC38 cells. (H) Measurement of tumor weight after subcutaneous injection. (I) CD8 immunohistochemistry in murine tumors. Scale bars, 200 µm. Data are presented as mean ± SEM; *p < 0.05; ***p < 0.001; one‐way ANOVA followed by Dunnett's multiple comparisons.

To further verify the tumor‐promoting effect of C1orf35 in vivo, nude mice were subcutaneously injected with HCT116 cells stably expressing shNC (control), shC1orf35‐1, and shC1orf35‐2. The results demonstrated that knockdown of C1orf35 significantly reduced tumor growth rate (Figure 8A,B). Immunohistochemical staining of endpoint tumor samples revealed that the positive areas of Ki‐67, C1orf35, and PYCR2 were significantly higher in the shNC group compared to the two knockdown groups. Moreover, the expression level of C1orf35 was positively correlated with Ki‐67 signal intensity, further confirming the positive regulatory effect of C1orf35 on cell proliferation (Figure 8C–F). To fully validate the role of C1orf35 in immune evasion, experiments in immunocompetent mouse models were executed. Subcutaneous implantation revealed that tumors derived from C1orf35‐knockdown MC38 cells (shmmtag2) exhibited markedly impaired growth and formed significantly smaller masses compared to those from control shNC cells (Figure 8G,H). Importantly, immunohistochemical analysis of the TME showed a notable increase in CD8^+^ T‐cell infiltration within shmmtag2 tumors relative to shNC controls (Figure 8I). These in vivo findings collectively demonstrate that C1orf35 knockdown suppresses tumor growth and suggest that its oncogenic function may be linked to the exclusion or functional suppression of CD8^+^ T cells.

## 3 Discussion

CRC is a molecularly heterogeneous disease driven by complex interactions among multiple genes and pathways. Its initiation, progression, and metastatic dissemination are all linked to the systemic dysregulation of signaling networks. A comprehensive understanding of these dysregulated pathways is crucial not only for early and accurate diagnosis but also for rational theoretical strategies, particularly in the era of combination targeted and immunotherapy [5, 10, 18, 19, 20]. Against this backdrop, our integrated analysis of TCGA, GEO, and tissue‐microarray data identified C1orf35 as a previously underappreciated oncoprotein. We found it to be highly expressed in CRC, with its levels strongly correlating with advanced tumor stage and unfavorable clinical outcomes. Functionally, C1orf35 serves as an independent prognostic factor for shorter OS, DSS, and PFI. Its high diagnostic accuracy (AUC = 0.938) further underscores its clinical potential. While prior studies implicated C1orf35 in other malignancies [8, 11, 21, 22], our in vitro and in vivo experiments provide definitive functional evidence in CRC, demonstrating that C1orf35 knockdown potently inhibits tumor cell proliferation, migration, and subcutaneous tumor growth. Targeting C1orf35 significantly suppresses tumor aggressiveness and extends survival, underlining its potential as both a prognostic biomarker and a therapeutic target.

Our pathway enrichment analyses revealed that high C1orf35 expression is linked to oxidative phosphorylation and DNA replication—critical processes that support energy production and rapid proliferation in cancer cells. PYCR2, a key enzyme in proline biosynthesis, plays a pivotal role in maintaining redox homeostasis and mitochondrial function, and has been implicated in tumor growth and metastasis [14]. In vivo, C1orf35 knockdown significantly inhibited tumor growth in nude mice, accompanied by reduced expression of Ki‐67 and PYCR2. Previous studies have indicated that high PYCR2 expression is associated with poor prognosis in patients with CRC [15, 16]. We further discovered that C1orf35 functions as a transcriptional regulator by binding to and activating the c‐MYC promoter, consistent with its reported role in other cancers [8]. Our study elucidates a pivotal C1orf35–c‐MYC–PYCR2 signaling axis that drives CRC progression through integrated metabolic reprogramming.

Most critically, our findings extend this model beyond cell‐intrinsic growth control to reveal a profound role in reshaping of the tumor immune microenvironment, defining a coherent “metabolism‐immune” regulatory mechanism. We demonstrate that the C1orf35/c‐MYC‐induced upregulation of PYCR2 is functionally linked to the suppression of anti‐tumor immunity. Multi‐algorithm immune infiltration analyses and multiplex immunofluorescence consistently revealed that high C1orf35 expression correlates with a significantly depleted immune landscape, characterized by markedly reduced infiltration of cytotoxic CD8^+^ T cells. Given that increased CD8^+^ T‐cell exhaustion (Tex) is strongly associated with poor patient prognosis in CRC [12, 23, 24, 25], this immunosuppressive phenotype is clinically significant. Previous reports have also shown that PYCR family molecules are enriched in various cancer signaling pathways and significantly associated with immune infiltration, tumor mutation burden (TMB) and microsatellite instability (MSI) [26], which aligns with the functional enrichment and immunosuppressive phenotype observed in our study. In our study, C1orf35‐overexpressing CRC cells directly impaired CD8^+^ T‐cell activation and cytokine production in co‐culture assays. This establishes a causal link between the tumor‐intrinsic C1orf35–PYCR2 metabolic pathway and extrinsic immune suppression. The negative spatial relationship between C1orf35 protein and CD8^+^ T‐cell density within human tumors, which intensifies with advancing stage, strongly supports the model that C1orf35, via c‐MYC and PYCR2, fosters an immune‐evasive niche. Based on our findings that C1orf35 enhances tumor immune evasion via the c‐Myc/PYCR2 axis, we propose two non‐mutually exclusive mechanistic directions to explain the observed suppression of CD8+ T‐cell function. First, the transcriptional activation of c‐Myc by C1orf35 may directly repress the expression of key T‐cell‐recruiting chemokines, such as CCL5, CXCL9, and CXCL10, within the TME. This would create an immunologically “cold” tumor by drastically reducing the chemotactic signals necessary for CD8+ T‐cell infiltration. Second, independent of or synergistic with the chemokine axis, the upregulation of PYCR2—a critical enzyme in proline metabolism—could initiate tumor cell‐intrinsic metabolic reprogramming. This reprogramming may lead to the depletion of essential nutrients or the accumulation of immunosuppressive metabolites (e.g., specific redox agents or kynurenine pathway products) in the local microenvironment, thereby directly impairing the survival, proliferation or effector functions of CD8^+^ T cells.

These insights position the C1orf35–c‐MYC–PYCR2 axis as a promising multi‐faceted therapeutic target. Its well‐defined components offer multiple avenues for pharmacological intervention, including direct inhibition of C1orf35's transcriptional activity or targeting of downstream effectors like PYCR2. Furthermore, considering the established efficacy of immune checkpoint inhibitors (ICIs) in improving CRC outcomes, any novel target capable of synergizing with or sensitizing tumors to existing immunotherapies holds significant clinical potential. Systematically elucidating the key role of C1orf35 in the TIME not only addresses a knowledge gap regarding its immune regulatory functions in CRC but also identifies it as a crucial player in immune microenvironment remodeling. This study represents the first comprehensive investigation into the oncogenic activity and immune regulatory functions of C1orf35 in CRC. Given its clear genomic location, C1orf35's targeted intervention offers both a theoretical foundation and practical advantages. In the TME, “de‐exhaustion” of CD8^+^ T cells is considered a core mechanism underlying the efficacy of ICIs, while T‐cell exhaustion is a key pathway for ICB resistance [27, 28, 29, 30, 31, 32, 33]. Therefore, high expression of C1orf35 associated with T‐cell exhaustion could effectively predict the efficacy of immunotherapy, with C1orf35 inhibitors combined with PD‐1/PD‐L1 antibodies expected to reverse immune suppression and significantly enhance the therapeutic effect of ICB [34, 35, 36, 37, 38, 39, 40, 41]. Our findings highlight its high diagnostic sensitivity and pathway‐specific activities, offering a novel perspective for the development of immune‐targeted therapeutic strategies in CRC and potentially other malignancies.

While this study establishes a novel role for C1orf35 in driving CRC progression and immune evasion, several limitations should be acknowledged. First, the precise molecular structure and full spectrum of interacting partners of the C1orf35 protein remain incompletely characterized, which may obscure additional functions beyond the c‐MYC–PYCR2 axis elucidated here. Second, although we demonstrate a clear spatial inverse correlation and a functional link in co‐culture, the exact in vivo sequence and relative contribution of the proposed dual mechanisms—direct chemokine repression versus metabolite‐mediated suppression of CD8^+^ T cells—require further dissection using more complex immune‐competent murine models or spatial multi‐omics. Third, the clinical translatability of targeting this axis, particularly the development of specific inhibitors against C1orf35, warrants future investigation. Addressing these limitations in subsequent studies will be crucial for validating the therapeutic potential of the C1orf35–c‐MYC–PYCR2 pathway and for translating these findings into clinical applications.

In conclusion, our integrated multi‐omics and functional data establish C1orf35 as a master regulator of CRC progression through a C1orf35–c‐MYC–PYCR2 axis. These findings advance our understanding of CRC biology and position C1orf35 as a promising candidate for developing diagnostic biomarkers and targeted therapies, particularly in combination with immunotherapeutic strategies.

## 4 Materials and Methods

### 4.1 Dataset Collection

RNA‐Seq data on gene expression and corresponding clinical information for pan cancer analysis encompassing major malignancies, including but not limited to COAD, BRCA, and LUAD (see Table S7 for the full list of 33 cancer types). Both normal and malignant tissues were obtained from TCGA (https://cancergenome.nih.gov/) and GEO (http://www.ncbi.nlm.nih.gov/geo). For the study, tissue microarrays and clinical specimens from CRC datasets were curated, including GSE89076, GSE22598, GSE110224, and GSE113513. All data were obtained from official, open‐access portals (GDC Data Portal for TCGA; NCBI GEO for GEO data). No special authorization was required. Data were accessed in October 2024.

### 4.2 Survival Analysis

Survival was evaluated using Kaplan–Meier (KM) curves with differences tested by the log‐rank test; patients were dichotomized into high‐ and low‐expression groups according to the cohort median of C1orf35. Associations between clinicopathological variables and survival were first examined by univariable Cox proportional‐hazards models, and variables with *p* < 0.10 were subsequently entered into a multivariable Cox model to identify independent prognostic predictors.

### 4.3 Enrichment Analysis

To investigate the biological role of C1orf35 in CRC, we performed differential expression analysis between low‐ and high‐C1orf35 groups using thresholds of |log_2_FC| > 1 and FDR < 0.05. Functional enrichment was carried out with the clusterProfiler R package (v3.6.3), including Gene Ontology (GO), Kyoto Encyclopedia of Genes and Genomes (KEGG), and gene set enrichment analysis (GSEA). GO annotations were examined at the levels of biological process (BP), cellular component (CC), and molecular function (MF). GSEA was applied to evaluate the statistical significance and concordance of pathway enrichment between phenotypes using predefined gene sets; enriched pathways were ranked by normalized enrichment score (NES) and adjusted *p*‐value. For KEGG analyses, we used the c2.cp.kegg.v2022.1.Hs.symbols.gmt gene set, and for GO analyses, we used c5.go.all.v2022.1.Hs.symbols.gmt (10,561 gene sets). Pathways with adjusted *p*‐value < 0.05 and FDR < 0.25 were considered significantly enriched.

### 4.4 Immune Infiltration Analyses

The ESTIMATE algorithm was used to assess the immune and stromal scores in CRC. Additionally, algorithms such as EPIC, MCPCOUNTER, QUANTISEQ, TIMER, XCELL, and TIP were utilized to analyze the correlation between C1orf35 expression and immune cell infiltration. Spearman's correlation analysis was employed to evaluate the relationship between C1orf35 expression and immune cells, while the Wilcoxon rank‐sum test was used to compare immune cell infiltration levels between low and high C1orf35 expression groups.

### 4.5 Patients and Clinical Samples

This study protocol was approved by the Ethical Review Board of Peking University People's Hospital. The study cohort comprised individuals diagnosed with CRC who underwent surgical resection at Peking University People's Hospital between July and September 2022. The CRC tissue microarray, consisting of 92 tumor samples and 45 normal tissue samples, was provided by Shanghai Outdo Biotech Company (Shanghai, China).

### 4.6 Immunohistochemistry and Multiplexed Immunofluorescence Staining Analysis

Tissue sections from formalin‐fixed, paraffin‐embedded (FFPE) samples were processed as follows. Deparaffinization was carried out with two changes of Histo‐Clear II. Sections were then rehydrated through a descending ethanol gradient. Following this, endogenous peroxidase activity was blocked and antigen retrieval was conducted. For immunohistochemistry and multiplexed immunofluorescence, sections were incubated with specific primary antibodies (C1orf35 (27930‐1‐AP, Proteintech), PYCR2 (17146‐1‐AP, Proteintech), CD8 (ab316778, Abcam), and Ki‐67 (ab15580, Abcam)) overnight at 4°C.

For immunohistochemistry (IHC), tissue sections were probed with horseradish‐peroxidase (HRP)‐conjugated secondary antibodies for 30 min at ambient temperature. Signal development was performed with 3,3′‐diaminobenzidine (DAB), followed by hematoxylin counterstaining.

Multiplex immunofluorescence was performed using the Opal 5‐Color Manual IHC Kit (PANOVUE). Following incubation with primary antibodies, the sections were subsequently subjected to sequential application of HRP‐conjugated secondary antibodies and tyramide signal amplification (TSA) fluorophores. Finally, nuclei were counterstained with DAPI, and slides were coverslipped with mounting medium.

Whole‐slide images were acquired using a NanoZoomer slide scanner and analyzed with ImageJ. Quantification of immunostaining was based on the mean intensity measured across three randomly selected, distinct fields per sample. In order to mitigate potential bias, the entire workflow—from staining and microscopy to image analysis—was performed under blinded conditions.

### 4.7 Cell Culture

HCT116 and SW480 colorectal carcinoma cells (ATCC) were routinely grown under standard conditions (37°C, 5% CO_2_, humidified air) in RPMI‐1640 medium supplemented with 10% FBS and 1% penicillin‐streptomycin (all from Gibco).

### 4.8 Quantitative Real‐Time PCR

Total RNA was isolated from HCT116 and SW480 cell lines with the RNAiso Plus reagent (Takara, Japan). RNA concentration and purity were determined spectrophotometrically before dissolution in RNase‐free water and storage at −80°C. First‐strand cDNA was then reverse‐transcribed from 1 µg of total RNA using the M‐MLV Reverse Transcriptase kit (Life Technologies, USA) with oligo(dT) primers. Gene‐specific primers (Table S8) were commercially synthesized by Sangon Biotech (China). Quantitative real‐time PCR amplifications were conducted in triplicate on an ABI StepOne Plus system (Applied Biosystems, USA) using the 2× SYBR‐Green qPCR SuperMix (High ROX), strictly adhering to the manufacturer's cycling protocols. Both biological and technical replicates were included to ensure data robustness.

### 4.9 Lentiviral Production and Transduction

shRNAs targeting human C1orf35 and PYCR2 were purchased from Gene Pharma company (Shanghai, China). Human C1orf35 shRNA (C1orf35‐Hum‐153, 5′‐GAGGACGTGAAGACTGACAAG‐3′, C1orf35‐Hum‐346, 5′‐GCTACAAGAACGTGAAGAAGC‐3′) and Human PYCR2 shRNA (PYCR‐Hum‐758, 5′‐GGCCUGCCUAUGCAUUCAUTT‐3′, PYCR ‐Hum‐307, 5′‐GCUCACAAGAUAAUAGCCATT‐3′) was cloned into the LV2N/Puro vector, then transfected into HCT116 and SW480 cells. We performed stable transfection by selecting cells in puromycin (10 µg/mL)‐supplemented DMEM. For lentiviral production, HEK‐293T cells were transfected with the psPAX2 packaging and pMD2G envelope plasmids (control: empty vector). After 48 h, supernatants containing lentiviral particles were collected. HCT116 or SW480 cells seeded in six‐well plates were transduced with the virus plus polybrene (Beyotime Biotechnology, China), followed by selection with 2 µg/mL puromycin (Amresco, USA). Transduction efficiency and C1orf35 expression levels were evaluated by western blot and real‐time PCR.

### 4.10 CCK‐8 Cell Viability Assay

We assessed cell proliferation using the Cell Counting Kit‐8 (CCK‐8; Dojindo, Japan) according to the manufacturer's instructions. Transfected cells were seeded into 96‐well plates at a density of 2000 cells per well and cultured for designated durations. At each time point, the optical density at 450 nm was measured with a Bio‐Rad microplate reader.

### 4.11 Colony Formation Assay

To assess clonogenic survival, cells were seeded into six‐well plates at a density of 1 × 10^3^ cells per well and maintained under standard culture conditions for a period of 10–15 days to allow colony formation. Colonies containing more than 50 cells were fixed with 4% paraformaldehyde, stained with 1% crystal violet for 10 min (G1062, Solarbio, China), and subsequently counted.

### 4.12 Transwell Assay

Cell migration and invasion were assessed using Transwell assays. Briefly, 2 × 10^4^ cells in serum‐free medium were seeded into the upper chamber of 24‐well Transwell inserts (3422, Corning, USA); inserts were pre‐coated with Matrigel (0827045, ABW, China) for invasion assays. The lower compartment was filled with 600 µL of complete growth medium, consisting of base medium with a 10% fetal bovine serum (FBS) supplement as a chemoattractant. After 36 h, cells that had migrated to or invaded the lower membrane surface were fixed with 4% paraformaldehyde and stained with 1% crystal violet. Images were acquired at 200× magnification using an Olympus IX53 microscope equipped with a DP73 camera, and six randomly selected fields per well were quantified.

### 4.13 Western Blotting Analysis

Total protein was isolated from tissue specimens using ice‐cold RIPA lysis buffer (P0013, Beyotime) supplemented with complete protease and phosphatase inhibitor cocktails. Protein concentrations were quantified to ensure equal loading. Subsequently, samples were resolved by 10% SDS‐PAGE and electro‐transferred onto polyvinylidene difluoride (PVDF) membranes (Millipore). Following a 1‐h block with 5% non‐fat dry milk in TBST at room temperature, the membranes were incubated overnight at 4°C with the following primary antibodies: anti‐C1orf35 (27930‐1‐AP, Proteintech) and anti‐β‐Actin (1:1000 dilution, 66009‐1‐Ig, Proteintech). After thorough washing, membranes were probed with appropriate horseradish peroxidase (HRP)‐conjugated secondary antibodies. Immunoreactive bands were finally visualized via enhanced chemiluminescence (ECL) substrate and imaged using a Bio‐Rad ChemiDoc system.

### 4.14 Patient‐Derived Organoid (PDO) Culture

Patient‐derived colorectal cancer organoids were established from fresh surgical specimens. Briefly, tumor tissues were minced and digested into single cells/small clusters, which were then embedded in basement membrane extract (BME). Organoids were cultured in Boygen Biotech's specialized CRC organoid growth medium, refreshed every 2–3 days, and routinely passaged. To investigate the functional role of C1orf35, established organoids were treated with recombinant human C1orf35 protein (source: Proteintech, Ag20769) at an optimized concentration of 50 ng/mL. The protein‐containing medium was replaced every 48 h for a treatment duration of 7 days. Organoid growth and morphological changes were monitored daily, and the proliferative response was quantitatively assessed by measuring cross‐sectional area from bright‐field images using ImageJ software.

### 4.15 Animal Experiments

All animal experiments were approved by the Ethics Committee of Peking University People's Hospital. We obtained BALB/c nude mice and C57BL/6 mice, aged 6 weeks, from the Animal Experiment Center of Peking University People's Hospital (Beijing, China). Prior to tumor inoculation, each animal was examined to confirm its health status and the absence of disease. Subsequently, we established xenograft models by administering a suspension of the 1 × 10^6^ HCT116 cells and 2 × 10^5^ CT26 cells subcutaneously into the right flank of BALB/c nude mice; similarly, the cells were engrafted into C57BL/6 mice using an identical protocol. Four weeks later, the animals were euthanized, and tumors were harvested. Tumor volumes and weights were recorded.

### 4.16 Statistical Analysis

Statistical analyses were performed based on data distribution. Normally distributed data were evaluated with parametric tests, and non‐normally distributed data with non‐parametric tests. Patient survival was evaluated using Kaplan–Meier curves, with differences assessed by the log‐rank test. Associations between clinicopathological variables and survival outcomes were first examined through univariate Cox proportional hazards regression. Variables showing an association with a threshold of *p < 0.1 were subsequently entered into a multivariate Cox model to identify independent prognostic factors. In all analyses, significance levels were denoted as: *p < 0.05, **p < 0.01, and ***p < 0.001.

## Author Contributions

**Shaosen Zhang**: conceptualization, methodology, resources, software, formal analysis, investigation, visualization, data curation, writing – original draft, writing – review and editing, funding acquisition. **Changjiang Yang**: conceptualization, methodology, resources, software, formal analysis, investigation, visualization, data curation, writing – original draft, writing – review and editing. **Xunye Xu**: investigation, writing – review and editing. **Lan Lan**: investigation, writing – review and editing. **Ziyi He**: investigation, writing – review and editing. **Jiaoting Chen**: investigation, writing – review and editing. **Caihong Wang**: funding acquisition, supervision, conceptualization, methodology, resources, software, formal analysis, investigation, visualization, data curation, writing – original draft, writing – review and editing. All authors have read and approved the final manuscript.

## Ethics Statement

All animal procedures were approved by the Institutional Animal Care and Use Committee (IACUC) (Animal Ethics Approval Number: 2025PHE116). All experimental protocols on patients were approved by the Ethics Committee of Peking University People's Hospital (reference number: 2025PHB611‐001) and were conducted in accordance with the ethical standards of the Declaration of Helsinki.

## Conflicts of Interest

The authors declare no conflicts of interest.

## Supporting information



Supporting Information: mco270707‐sup‐0001‐TableS1.xlsx"

**Table S2**: Relationship between C1orf35 Expression and Clinicopathological Characteristics in the TCGA Cohort.
**Table S3**: Univariate and multivariate analyses of OS.
**Table S4**: Univariate and Multivariate Analyses of DSS.
**Table S5**: Univariate and Multivariate Analyses of PFI.
**Table S6**: Correlation between C1orf35 Expression and Immune Infiltration in CRC.
**Table S7**: The 33 cancer types and their abbreviations from The Cancer Genome Atlas (TCGA) pan‐cancer cohort.
**Table S8**: Primer sequences for q‐RT‐PCR analysis.

## Data Availability

All data are available from the corresponding author upon reasonable request.
