# Supplementary material for: Chromosome 1 Open Reading Frame 35 Drives Colorectal Cancer Progression by Enhancing Tumor‐Intrinsic Proliferation and CD8+ T Cell Suppression
Source: MedComm (2020). 2026 Mar 28;7(4):e70707. doi: 10.1002/mco2.70707 (PMC13042507; doi:10.1002/mco2.70707)
Supplement: Supplementary file 2 — Table S2: Relationship between C1orf35 Expression and Clinicopathological Characteristics in the TCGA Cohort. Table S3: Univariate and multivariate analyses of OS. Table S4: Univariate and Multivariate Analyses of DSS. Table S5: Univariate and Multivariate Analyses of PFI. Table S6: Correlation between C1orf35 Expression and Immune Infiltration in CRC. Table S7: The 33 cancer types and their abbreviations from The Cancer Genome Atlas (TCGA) pan‐cancer cohort. Table S8: Primer sequences for q‐RT‐PCR analysis. [file MCO2-7-e70707-s001.docx]

**Chromosome 1 open reading frame 35 Drives Colorectal Cancer Progression by Enhancing Tumor-Intrinsic Proliferation and CD8^+^ T Cell Suppression**

Shaosen Zhang^1, 2, 5, *^, Changjiang Yang^3, 4, 5^, Xunye Xu^3, 4, 5^, Lan Lan^1, 2^, Ziyi He^1, 2^, Jiaoting Chen^1, 2^, Caihong Wang^3, 4, *^

^1^Department of Etiology and Carcinogenesis, National Cancer Center/National Clinical Research Center/Cancer Hospital, Chinese Academy of Medical Sciences (CAMS) and Peking Union Medical College (PUMC), Beijing 100021, China

^2^Key Laboratory of Cancer Genomic Biology, Chinese Academy of Medical Sciences and Peking Union Medical College, Beijing 100021, China

^3^Department of Gastroenterological Surgery, Peking University People’s Hospital, Beijing 100044, China

^4^Laboratory of Surgical Oncology, Beijing Key Laboratory of Colorectal Cancer Diagnosis and Treatment Research, Peking University People’s Hospital, Beijing 100044, China

^5^These authors contribute equally.

*Correspondence: zhangss@cicams.ac.cn (S.Z.); wangcaihong@pkuph.edu.cn (C.W.)

Running title: C1orf35 Drives CRC Tumor Growth and Immune Envasion

**Table S2**: Relationship between C1orf35 Expression and Clinicopathological Characteristics in the TCGA Cohort

| Characteristics | Low expression of C1orf35 | High expression of C1orf35 | P value |
| --- | --- | --- | --- |
| n | 322 | 322 |  |
| Pathologic T stage, n (%) |  |  | 0.020 |
| T1 | 13 (2%) | 7 (1.1%) |  |
| T2 | 64 (10%) | 47 (7.3%) |  |
| T3 | 215 (33.5%) | 221 (34.5%) |  |
| T4 | 27 (4.2%) | 47 (7.3%) |  |
| Pathologic N stage, n (%) |  |  | 0.003 |
| N0 | 205 (32%) | 163 (25.5%) |  |
| N1 | 65 (10.2%) | 88 (13.8%) |  |
| N2 | 49 (7.7%) | 70 (10.9%) |  |
| Pathologic M stage, n (%) |  |  | 0.005 |
| M0 | 243 (43.1%) | 232 (41.1%) |  |
| M1 | 31 (5.5%) | 58 (10.3%) |  |
| Pathologic stage, n (%) |  |  | < 0.001 |
| Stage I | 69 (11.1%) | 42 (6.7%) |  |
| Stage II | 130 (20.9%) | 108 (17.3%) |  |
| Stage III | 82 (13.2%) | 102 (16.4%) |  |
| Stage IV | 32 (5.1%) | 58 (9.3%) |  |
| Gender, n (%) |  |  | 0.236 |
| Female | 158 (24.5%) | 143 (22.2%) |  |
| Male | 164 (25.5%) | 179 (27.8%) |  |
| Age, n (%) |  |  | 0.111 |
| <= 65 | 128 (19.9%) | 148 (23%) |  |
| > 65 | 194 (30.1%) | 174 (27%) |  |

**Table S3**: Univariate and multivariate analyses of OS

| Characteristics | Total(N) | Univariate analysis | |  | Multivariate analysis | |
| --- | --- | --- | --- | --- | --- | --- |
|  |  | Hazard ratio (95% CI) | P value |  | Hazard ratio (95% CI) | P value |
| Pathologic T stage | 640 |  |  |  |  |  |
| T1&T2 | 131 | Reference |  |  | Reference |  |
| T3&T4 | 509 | 2.468 (1.327 - 4.589) | 0.004 |  | 2.164 (0.983 - 4.766) | 0.055 |
| Pathologic N stage | 639 |  |  |  |  |  |
| N0 | 367 | Reference |  |  | Reference |  |
| N1&N2 | 272 | 2.627 (1.831 - 3.769) | < 0.001 |  | 0.509 (0.194 - 1.333) | 0.169 |
| Pathologic M stage | 563 |  |  |  |  |  |
| M0 | 474 | Reference |  |  | Reference |  |
| M1 | 89 | 3.989 (2.684 - 5.929) | < 0.001 |  | 2.356 (1.460 - 3.804) | < 0.001 |
| Pathologic stage | 622 |  |  |  |  |  |
| Stage  I&Stage II | 348 | Reference |  |  | Reference |  |
| Stage III&Stage IV | 274 | 2.988 (2.042 - 4.372) | < 0.001 |  | 4.370 (1.490 - 12.813) | 0.007 |
| Gender | 643 |  |  |  |  |  |
| Female | 301 | Reference |  |  |  |  |
| Male | 342 | 1.054 (0.744 - 1.491) | 0.769 |  |  |  |
| Age | 643 |  |  |  |  |  |
| <= 65 | 276 | Reference |  |  | Reference |  |
| > 65 | 367 | 1.939 (1.320 - 2.849) | < 0.001 |  | 2.839 (1.824 - 4.421) | < 0.001 |
| C1orf35 | 643 |  |  |  |  |  |
| Low | 322 | Reference |  |  | Reference |  |
| High | 321 | 1.650 (1.151 - 2.366) | 0.006 |  | 1.506 (1.009 - 2.246) | 0.045 |

**Table S4**: Univariate and Multivariate Analyses of DSS

| Characteristics | Total(N) | Univariate analysis | |  | Multivariate analysis | |
| --- | --- | --- | --- | --- | --- | --- |
|  |  | Hazard ratio (95% CI) | P value |  | Hazard ratio (95% CI) | P value |
| Pathologic  T stage | 618 |  |  |  |  |  |
| T1&T2 | 129 | Reference |  |  | Reference |  |
| T3&T4 | 489 | 6.440 (2.029 - 20.441) | 0.002 |  | 2.735 (0.831 - 9.002) | 0.098 |
| Pathologic  N stage | 617 |  |  |  |  |  |
| N0 | 358 | Reference |  |  | Reference |  |
| N1&N2 | 259 | 4.119 (2.496 - 6.797) | < 0.001 |  | 0.657 (0.252 - 1.714) | 0.390 |
| Pathologic  M stage | 542 |  |  |  |  |  |
| M0 | 455 | Reference |  |  | Reference |  |
| M1 | 87 | 7.471 (4.647 - 12.012) | < 0.001 |  | 3.800 (2.118 - 6.818) | < 0.001 |
| Pathologic stage | 601 |  |  |  |  |  |
| Stage I&Stage II | 339 | Reference |  |  | Reference |  |
| Stage III&Stage IV | 262 | 5.716 (3.240 - 10.083) | < 0.001 |  | 3.698 (1.124 - 12.167) | 0.031 |
| Gender | 621 |  |  |  |  |  |
| Female | 290 | Reference |  |  |  |  |
| Male | 331 | 1.207 (0.769 - 1.895) | 0.412 |  |  |  |
| Age | 621 |  |  |  |  |  |
| <= 65 | 273 | Reference |  |  |  |  |
| > 65 | 348 | 1.421 (0.894 - 2.257) | 0.137 |  |  |  |
| C1orf35 | 621 |  |  |  |  |  |
| Low | 316 | Reference |  |  | Reference |  |
| High | 305 | 2.317 (1.431 - 3.752) | < 0.001 |  | 1.511 (0.910 - 2.510) | 0.111 |

**Table S5**: Univariate and Multivariate Analyses of PFI

| Characteristics | Total(N) | Univariate analysis | |  | Multivariate analysis | |
| --- | --- | --- | --- | --- | --- | --- |
|  |  | Hazard ratio (95% CI) | P value |  | Hazard ratio (95% CI) | P value |
| Pathologic  T stage | 640 |  |  |  |  |  |
| T1&T2 | 131 | Reference |  |  | Reference |  |
| T3&T4 | 509 | 3.198 (1.814 - 5.636) | < 0.001 |  | 1.831 (1.011 - 3.317) | 0.046 |
| Pathologic  N stage | 639 |  |  |  |  |  |
| N0 | 367 | Reference |  |  | Reference |  |
| N1&N2 | 272 | 2.624 (1.916 - 3.592) | < 0.001 |  | 1.017 (0.432 - 2.395) | 0.968 |
| Pathologic  M stage | 563 |  |  |  |  |  |
| M0 | 474 | Reference |  |  | Reference |  |
| M1 | 89 | 5.577 (3.945 - 7.884) | < 0.001 |  | 4.405 (2.825 - 6.868) | < 0.001 |
| Pathologic stage | 622 |  |  |  |  |  |
| Stage I&Stage II | 348 | Reference |  |  | Reference |  |
| Stage III&Stage IV | 274 | 2.924 (2.115 - 4.044) | < 0.001 |  | 1.251 (0.482 - 3.248) | 0.646 |
| Gender | 643 |  |  |  |  |  |
| Female | 301 | Reference |  |  |  |  |
| Male | 342 | 1.217 (0.892 - 1.660) | 0.216 |  |  |  |
| Age | 643 |  |  |  |  |  |
| <= 65 | 276 | Reference |  |  |  |  |
| > 65 | 367 | 1.006 (0.737 - 1.371) | 0.972 |  |  |  |
| C1orf35 | 643 |  |  |  |  |  |
| Low | 322 | Reference |  |  | Reference |  |
| High | 321 | 1.743 (1.272 - 2.390) | < 0.001 |  | 1.307 (0.934 - 1.828) | 0.118 |

**Table S6**: Correlation between C1orf35 Expression and Immune Infiltration in CRC

|  | ID | Spearman | P value |
| --- | --- | --- | --- |
| EPIC | B cell | -0.234677155 | 3.33061E-09 |
|  | T cell CD4+ | -0.234374143 | 3.49333E-09 |
|  | T cell CD8+ | -0.076501787 | 0.056932878 |
|  | Endothelial cell | -0.08128321 | 0.04305253 |
|  | Macrophage | -0.129975283 | 0.001180308 |
|  | NK cell | -0.036002285 | 0.370824098 |
|  | uncharacterized cell | 0.199990086 | 5.15455E-07 |
| MCPCOUNTER | T cell | -0.080694986 | 0.044589065 |
|  | T cell CD8+ | -0.035466728 | 0.377987932 |
|  | cytotoxicity score | -0.144797635 | 0.000297766 |
|  | NK cell | -0.165282776 | 3.53905E-05 |
|  | B cell | -0.200066972 | 5.10194E-07 |
|  | Monocyte | -0.200528866 | 4.7966E-07 |
|  | Macrophage/Monocyte | -0.200528866 | 4.7966E-07 |
|  | Myeloid dendritic cell | -0.275854858 | 2.73007E-12 |
|  | Neutrophil | -0.101301221 | 0.011609891 |
|  | Endothelial cell | -0.134578464 | 0.000780811 |
| QUANTISEQ | B cell | -0.136909306 | 0.000630267 |
|  | Macrophage M1 | -0.22153028 | 2.48479E-08 |
|  | Macrophage M2 | -0.103510021 | 0.009905565 |
|  | Monocyte | 0.056928816 | 0.156832932 |
|  | Neutrophil | -0.27827286 | 1.7289E-12 |
|  | NK cell | 0.046488391 | 0.247746351 |
|  | T cell CD4+  (non-regulatory) | -0.097645974 | 0.015004376 |
|  | T cell CD8+ | -0.099218512 | 0.013449609 |
|  | T cell regulatory (Tregs) | -0.307742634 | 4.56768E-15 |
|  | Myeloid dendritic cell | -0.031236929 | 0.437505088 |
|  | uncharacterized cell | 0.347217957 | 5.25259E-19 |
| TIMER | B cell | -0.166440883 | 3.1126E-05 |
|  | T cell CD4+ | -0.032770361 | 0.415331342 |
|  | T cell CD8+ | -0.33533806 | 9.26326E-18 |
|  | Neutrophil | -0.210312249 | 1.25437E-07 |
|  | Macrophage | -0.162958423 | 4.56745E-05 |
|  | Myeloid dendritic cell | -0.263996539 | 2.40509E-11 |

**Table S7**: The 33 cancer types and their abbreviations from The Cancer Genome Atlas (TCGA) pan-cancer cohort

| Full Name | Abbreviation |
| --- | --- |
| Adrenocortical carcinoma | ACC |
| Bladder Urothelial Carcinoma | BLCA |
| Breast invasive carcinoma | BRCA |
| Cervical squamous cell carcinoma and endocervical adenocarcinoma | CESC |
| Cholangiocarcinoma | CHOL |
| Colon adenocarcinoma | COAD |
| Lymphoid Neoplasm Diffuse Large B-cell Lymphoma | DLBC |
| Esophageal carcinoma | ESCA |
| Glioblastoma multiforme | GBM |
| Head and Neck squamous cell carcinoma | HNSC |
| Kidney Chromophobe | KICH |
| Kidney renal clear cell carcinoma | KIRC |
| Kidney renal papillary cell carcinoma | KIRP |
| Acute Myeloid Leukemia | LAML |
| Brain Lower Grade Glioma | LGG |
| Liver hepatocellular carcinoma | LIHC |
| Lung adenocarcinoma | LUAD |
| Lung squamous cell carcinoma | LUSC |
| Mesothelioma | MESO |
| Ovarian serous cystadenocarcinoma | OV |
| Pancreatic adenocarcinoma | PAAD |
| Pheochromocytoma and Paraganglioma | PCPG |
| Prostate adenocarcinoma | PRAD |
| Rectum adenocarcinoma | READ |
| Sarcoma | SARC |
| Skin Cutaneous Melanoma | SKCM |
| Stomach adenocarcinoma | STAD |
| Testicular Germ Cell Tumors | TGCT |
| Thyroid carcinoma | THCA |
| Thymoma | THYM |
| Uterine Corpus Endometrial Carcinoma | UCEC |
| Uterine Carcinosarcoma | UCS |
| Uveal Melanoma | UVM |

**Table S8**: Primer sequences for q-RT-PCR analysis.

| Gene | Forward (5'-3') | Reverse (5'-3') |
| --- | --- | --- |
| C1orf35 | GCGGAGGATCAGACGGAAAG | TCTCAGGAGATGTGGGAGAGGAG |
| c-MYC | CCTGGTGCTCCATGAGGAGA | CTCCAGCAGAAGGTGATCCAGA |
| PYCR2 | GCCAGCTCCCCAGAAATGAA | GTTGGTCATGCAGCGAATCA |
